# Organic carbon burial during OAE2 driven by changes in the locus of organic matter sulfurization

**DOI:** 10.1038/s41467-018-05943-6

**Published:** 2018-08-24

**Authors:** Morgan Reed Raven, David A. Fike, Maya L. Gomes, Samuel M. Webb, Alexander S. Bradley, Harry-Luke O. McClelland

**Affiliations:** 1 Department of Earth and Planetary Sciences, Washington University in St Louis, St Louis, MO 63130 USA; 2 Department of Earth and Planetary Sciences, Johns Hopkins University, Baltimore, MD 21218 USA; 3 Stanford Synchrotron Radiation Lightsource, Stanford University, Menlo Park, CA 94025 USA; 4 Department of Earth and Planetary Sciences, Weizmann Institute of Science, Rehovot, Israel

## Abstract

Ocean Anoxic Event 2 (OAE2) was a period of dramatic disruption to the global carbon cycle when massive amounts of organic matter (OM) were buried in marine sediments via complex and controversial mechanisms. Here we investigate the role of OM sulfurization, which makes OM less available for microbial respiration, in driving variable OM preservation in OAE2 sedimentary strata from Pont d’Issole (France). We find correlations between the concentration, S:C ratio, S-isotope composition, and sulfur speciation of OM suggesting that sulfurization facilitated changes in carbon burial at this site as the chemocline moved in and out of the sediments during deposition. These patterns are reproduced by a simple model, suggesting that small changes in primary productivity could drive large changes in local OM burial in environments poised near a critical redox threshold. This amplifying mechanism may be central to understanding the magnitude of global carbon cycle response to environmental perturbations.

## Abstract (web summary)

The mechanisms responsible for the burial of vast quantities of organic matter during Ocean Anoxic Event remain unclear. Here, the authors combine biogeochemical analysis and modeling and show that sulfurization could play a critical role in facilitating globally elevated burial of organic matter.

## Introduction

Organic matter (OM) burial in marine sediments is a central flux in the global carbon cycle, impacting both global biogeochemistry and climate. It tends to be concentrated in shallow, productive, and relatively O_2_-depleted environments^1–3^, where microbial communities utilize a variety of nitrogen-based, metal-based, and sulfur-based metabolisms to gain energy. Still, it remains difficult to predict rates of OM burial in marine sediments and how those rates might respond to environmental changes, with implications for modern climate change as well as many key intervals of biogeochemical disruption in Earth history.

Only a very small fraction of the carbon that primary producers fix in the surface ocean is buried in underlying sediments, while the vast majority is remineralized by heterotrophic organisms. In the presence of abundant O_2_, remineralization is generally fast and thorough, leaving organic carbon concentrations in open-ocean sediments below 0.5 wt%^4^. In anoxic environments, which can produce rocks with up to 52 wt% organic carbon^5^, a larger proportion of OM is resistant to degradation than in oxic environments^6,7^ even though rates of microbial heterotrophy can be similar to those in oxic systems^8^. OM may be more effectively recalcitrant under O_2_-limited conditions for several reasons, including reduced efficacy of exoenzymes^9^, lack of macrofaunal bioturbation^10^, thermodynamic inhibition^11^, and/or transformation of OM due to abiotic reactions with sulfide, called ‘sulfurization’^12^. It is this latter process that we focus on in this work. Sulfurization is thought to limit OM bioavailability by removing energy-yielding functional groups, increasing OM molecular weight, and potentially making structures incompatible with exoenzyme degradation^13^. OM sulfurization thus provides a potentially strong mechanistic link between sedimentary sulfur and carbon burial^14,15^.

Although sulfurization reactions are themselves abiotic, they depend on a source of sulfide from microbial processes. In the absence of O_2_, sulfate-reducing microorganisms generate hydrogen sulfide (H_2_S) and other reduced sulfur species to gain energy from OM. These compounds can react with OM, increasing its S:C ratio from values typical of planktonic/microbial biomass (0.6–1.0%) to as much as 8%^16,17^. Abiotic sulfur addition is generally associated with oxygen loss, suggesting that the sulfide substitutes for hydroxyl groups^18^ and is added across double bonds^19^. Substantial amounts of this “abiogenic S_org_” form in sinking particles and surface sediments, sometimes even prior to the detection of sulfide in pore waters^20,21^. Because sulfurization in these environments can impact a relatively large and reactive pool of OM, it can potentially drive major changes in OM preservation^22,23^. A second category of sulfurization reactions can also occur gradually, over thousands of years in strongly reducing sediments^24^.

In this study, we investigate the roles of rapid and gradual OM sulfurization in controlling OM burial fluxes across an interval of major environmental redox change, Ocean Anoxic Event 2 (OAE2, ~94 million years ago (Ma)). Specifically, we investigate a section from the Cenomanian–Turonian boundary at Pont d’Issole, southeastern France, that has been correlated with the Eastbourne OAE2 European reference section and other globally distributed OAE2 sections based on biostratigraphy and carbon isotope chemostratigraphy^25^. Unlike most sites at which sulfurization has been identified previously^5,17,26^, the Pont d’Issole section preserves an expanded record of moderate and variable OM preservation (0.06–3.0 wt% TOC), allowing us to explore how changes in local redox state impact the location, rate, and intensity of OM sulfurization and, by extension, carbon burial.

## Results

### Study site

**Fig. 1:**
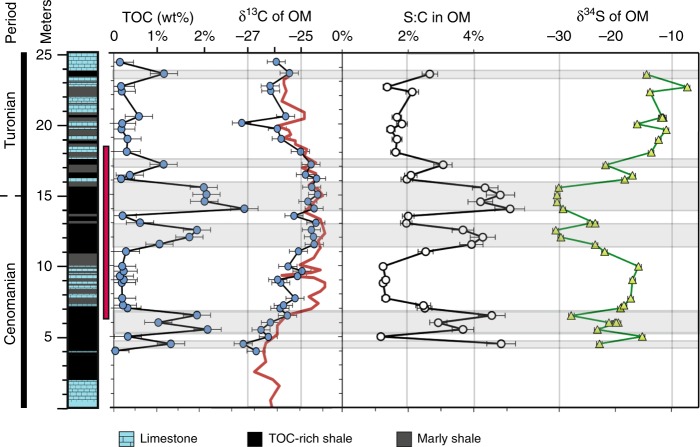
Stratigraphic profiles of organic sulfur and carbon geochemistry at Pont d’Issole. Total organic carbon (TOC) concentrations, δ^13^C values (in permil (‰) vs. the VPDB standard), S:C (mol/mol) ratios, and δ^34^S values (in ‰ vs. the VCDT standard) show clear and coherent variations with depth. Stratigraphy at left is from^25,29^ and the red bar indicates the positive C-isotope excursion as in^25^. Shaded bands highlight intervals with TOC concentrations >1.0%. For organic carbon δ^13^C profiles, the red line shows data from^25^, while blue symbols are new data for our samples. Error bars indicate the reproducibility of representative external replicates (*n* ≥ 3). Where OM is abundant, it is associated with relatively high molar S:C ratios and low δ^34^S values

The Pont d’Issole section is exposed along the Issole River 500 m southeast of the village of La Bâtie Thorame‐Basse, Alpe de Haute Provence in France^27^. The sediments were deposited in the deeper part of the Vocontian Basin, a gulf of the Tethys Ocean, with an estimated water depth of several hundred meters^27,28^. Samples were collected in July 2011^29^. Age control is based on biostratigraphy and correlation of the carbonate δ^13^C record to the reference section at Eastbourne^25,30^. This Cenomanian-Turonian Boundary section has several distinctive organic-rich layers, in which benthic foraminifera are absent and fine-scale laminations of OM and detritus-rich material alternate with calcareous mudstones (Fig.1). The remainder of the section is characterized by interbedded limestones and marls, which contain both planktic and benthic foraminifera^27^. Previous work on this section generated S-isotope profiles for pyrite and carbonate-associated sulfate^29^ as well as bio and chemostratigraphy, including C-isotope ratios^25,31^.

### Organic S geochemical profiles

OAE2 strata from Pont d’Issole consist of alternating bands of limestones, marly shales and moderately TOC-rich shales (Fig. 1). The highest TOC concentrations (up to 3.1 wt%) are found in the dark shales with occasional limestone beds that lie between 11.5 and 15.5 m and record the some of the most ^13^C-enriched values of the positive C-isotope excursion. Narrower layers of TOC-rich shales appear throughout the section, notably at the culmination of the C-isotope excursion at 17.1 m and in interbedded deposits from 4 to 7 m. These shales contain up to 3.1 wt% organic C—substantially elevated over typical oxic marine sediments (~0.34 wt%^4^) but much lower than OAE2 sediments from other environments, where maximum TOC concentrations can approach 30 wt%^32^. TOC concentrations in the limestone–marl layers are ~0.2 wt%, near typical modern, oxic marine sediment values^4^. Throughout this discussion, “TOC” and “S_org_” refer to the residual organics in sediment after solvent extraction and chromium reduction (i.e., “kerogens”) and exclude solvent-extractable organics (i.e., “bitumens”), a relatively small pool equivalent to 0.7–3.4% of the S in kerogens (Supplementary Table 1).

Our samples reproduce the global excursion in C-isotope ratios that is seen in both organic carbon at this site and elsewhere (see ref.^25^, red line in Fig. 1). OM is ^13^C-enriched through much of the section from 5 to 18 m, above which it shifts back toward pre-event δ^13^C values. The OAE2 positive C isotope excursion is seen globally and is thought to result from a massive increase in the global burial flux of ^13^C-depleted organic carbon over this interval, a perturbation that left the marine dissolved inorganic carbon pool relatively ^13^C-enriched^33^. TOC-rich shales were deposited at Pont d’Issole for some, but not all, of that interval. The duration of the OAE2 C-isotope excursion is estimated to be approximately 440,000–820,000 years based on astronomical tuning^34–36^.

Molar S:C ratios in OM in these strata generally parallel TOC concentrations (Fig. 1). TOC-rich shales at the peak of local OAE2 deposition reach a S:C ratio of 5.1% (mol/mol). In OM-lean rocks (~0.2 wt% TOC), S:C ratios are much lower, near 1.2%. For comparison, fresh marine biomass typically has a molar S:C ratio between 0.6 and 1%^20^. The S:C ratio of OM does not co-vary with the N:C ratio of OM, as might be expected if the primary source of organic S were complete amino acids (Supplementary Table 1).

Like S:C, the S-isotope composition of OM varies coherently across the section, ranging from –30.6‰ in a TOC-rich shale at 12.5 m to –7.2‰ in a limestone-marl at 22.6 m. These patterns mirror trends in the S:C ratio of OM with stratigraphic height, with low δ^34^S values associated with higher TOC concentrations and higher S:C ratios, and vice versa (Fig. 1). The association between more strongly ^34^S-depleted values and TOC-rich intervals holds for the interbedded shales and carbonates around the onset of the event (4–7 m) as well as the thicker TOC-rich shales during the core of the OAE.

### Organic S redox character (X-ray absorption spectra (XAS))

**Fig. 2:**
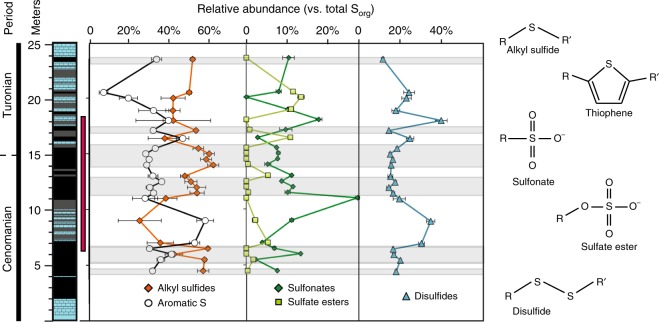
Relative abundance of organic sulfur in various redox states with depth. The red bar at left indicates the duration of the OAE2 C-isotope excursion, and shaded bands indicate intervals with TOC concentrations >1 wt%. Error bars represent uncertainties from spectrum fitting via SIXPACK (1 s.d., Supplementary Table 2). Low-TOC rocks tend to be richer in aromatic S (open circles) while high-TOC rocks contain substantial amounts of alkyl sulfides (orange diamonds). Representative S_org_ structures are shown on the right

XAS indicate that S_org_ in Pont d’Issole rocks primarily consists of reduced forms, predominantly alkyl sulfides and aromatic S, which encompases thiophenes, benzothiophenes, naphthalenes, and aryl disulfides (Fig. 2). Together, these reduced S pools make up 65–94% of total S_org_, with the remainder composed of “oxidized” S_org_: sulfonates, sulfoxides, and sulfate esters (example spectra provided in Supplementary Figure 1). There is no spectral signature for pyrite in these powders, indicating that chemical extractions to remove this phase were effectively complete. The fractional abundances of each S_org_ moiety and the uncertainties associated with spectrum fitting are summarized in Supplementary Table 2.

The redox character of S_org_ varies with the abundances of TOC and S_org_ across OAE2. The TOC-rich (>1 wt%) rocks deposited at the center of OAE2 C-isotope excursion (11.5–15.5 m) have a consistent redox speciation, containing 57 ± 3% monosulfides, 32 ± 3% aromatic S, 17 ± 5% disulfides, 8 ± 2% sulfonates, and 3 ± 1% sulfoxides (mean ± 1σ). In low-TOC rocks, S_org_ contains larger proportions of sulfonates, sulfate esters, and aromatics at the expense of alkyl sulfides.

## Discussion

**Fig. 3:**
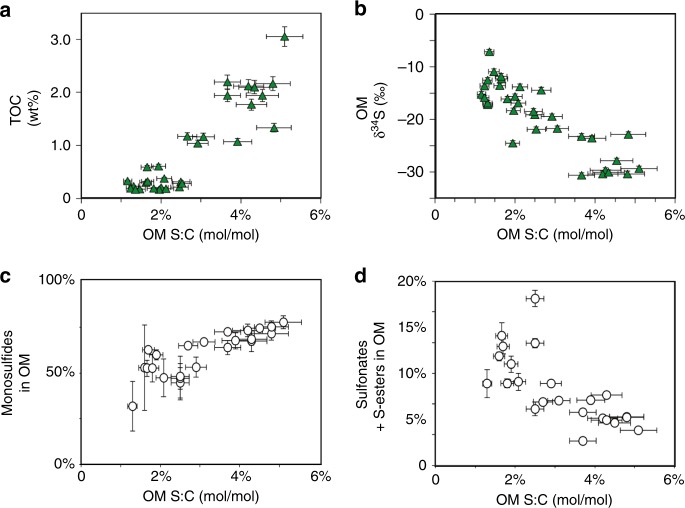
Relationships between the molar S:C ratio of OM and other S_org_ characteristics. **a** TOC concentration, **b** OM δ^34^S value, and the relative abundances of **c** monosulfides and **d** oxidized (sulfonate + sulfate ester) S_org_ in OM. Relative uncertainties (1 s.d.) on S:C ratios are calculated from propagation of error from the reproducibility of external replicate (*n* ≥ 3) tests. Uncertainties for XAS data are from spectrum fitting (1 s.d., Supplementary Table 2)

The abundance, S:C ratio, S-isotope composition, and redox character of OM in Pont d’Issole rocks vary across OAE2 in ways that support a central role for abiotic sulfurization in net OM preservation. OM S:C ratios provide an indicator of the intensity of sulfurization^5^, which is the only known mechanism (other than mixing with detrital material from a strongly sulfurized deposit) for increasing bulk OM S:C ratios above those of biomass, which has a maximum reported S:C ratio of ~2%^20,37^. In contrast, OM breakdown and remineralization by heterotrophs generally reduces the S:C ratio of fresh biomass in the environment, apparently due to preferential utilization of proteins by microbial communities^7^. The molar S:C ratios of Pont d’Issole kerogens are strongly positively correlated with TOC concentrations on both a whole–rock (*R*^2^ = 0.81, Fig. 3) and carbonate-free basis (*R*^2^ = 0.73, Supplementary Table 1), indicating that the most TOC-rich rocks have been the most intensely sulfurized.

Extensive OM sulfurization has been described previously for two other OAE2 sections (Tarfaya Basin, Morocco and Demerara Rise, offshore Brazil) with much higher TOC concentrations^17,26^, and for the Kimmeridge Clay (Jurassic), which is also extremely OM-rich (≤52 wt%)^14,22^. Unlike these sites, Pont d’Issole rocks contain moderate amounts of OM (0.06–3.0 wt% TOC), making them a potential record of the transitions between high and low OM burial regimes.

The S-isotope composition of OM at Pont d’Issole can be used to evaluate changes in the position of the chemocline, allowing us to specify the location and timing of OM sulfurization in the paleoenvironment. Stratigraphic variations in the δ^34^S value of pyrite are frequently explained in terms of relatively “open” versus “closed” system deposition with respect to the microbial consumption of dissolved sulfate^38^, and this model is equally applicable to abiogenic S_org_. Simplistically, in a more “open” sedimentary system, the δ^34^S value of S_org_ reflects the δ^34^S value of seawater sulfate minus the (typically large) fractionation associated with microbial sulfate reduction. In a more diffusively restricted or “closed” sedimentary system, sulfate becomes increasingly ^34^S-enriched as it is consumed by microbial sulfate reduction, and gradual sulfurization can generate S_org_ with a correspondingly more ^34^S-enriched composition. As shown in Fig. 3b, the abundant S_org_ in relatively TOC-rich Pont d’Issole rocks tends to be strongly ^34^S-depleted, while S_org_ in low-TOC Pont d’Issole rocks is relatively ^34^S-enriched. This negative correlation is consistent with the locus of S_org_ formation moving in and out of deeper, more diffusively restricted sediments as well as with lithologic evidence for anoxia at the sediment-water interface during periods of open-system OM sulfurization. During the deposition of TOC-rich shales at Pont d’Issole, we conclude that sulfide was available in an open system (e.g., the water column or sediment-water interface), facilitating rapid reactions between polysulfides and abundant, reactive OM and generating relatively large amounts of S-rich OM with a strongly ^34^S-depleted composition. During the deposition of more TOC-lean limestones, sulfurization was limited to deeper within the sediments, yielding more ^34^S-enriched products. To have developed a closed-system signature in this steadily accumulating marine system, OM sulfurization reactions would have occurred at much lower rates (e.g., over tens to thousands of years) than in particles.

Two distinct regimes of OM sulfurization have similarly been observed in experiments and modern environments: rapid sulfurization between polysulfides and fresh OM on timescales of days, which may dominate S_org_ formation in anoxic environments near redox interfaces^21^, and gradual sulfurization between sulfide and degraded OM in deeper, more reducing sediments on timescales of thousands of years^21^. Sediments from Pont d’Issole span a critical transition zone between these regimes, recording how marginal changes in O_2_ availability impact local carbon burial. Changes in the intensity of abiotic sulfurization were closely linked with changes in OM burial at Pont d’Issole across OAE2, including when the chemocline was sufficiently below the sediment-water interface to restrict the availability of sulfate, yielding more ^34^S-enriched OM. At this site, sulfurization thus appears to be a primary control on the response of carbon burial to marginal changes in chemocline position across the transition from oxic to sub-oxic or anoxic bottom water. This indicates that the range of environments potentially impacted by OM sulfurization extends beyond extremely O_2_-limited basins to include many sub-oxic environments, including OM-rich coastal, oxygen minimum zone and shelf sites.

Organic S at sites with fluctuating redox conditions like Pont d’Issole is likely to comprise a mixture of both rapid and gradual sulfurization products, plus any surviving biomass and detritus. To assess the contributions of these various potential sources of S_org_, we use XAS to quantitatively deconvolve the contributions of different S_org_ moieties to preserved OM across the full stratigraphic profile at Pont d’Issole. XAS has been used widely to understand S_org_ in soils^39,40^ and individual kerogens^41,42^, but there are relatively few examples of marine S_org_ redox speciation data in stratigraphic context^16,43,44^.

S_org_ in Pont d’Issole OAE2 rocks is dominated by reduced forms (nominal oxidation state of 0^45^), but the balance between aliphatic and aromatic reduced S_org_ shifts with sulfurization intensity. In the most TOC-rich shales (11.5–15.5 m), aliphatic and aromatic S_org_ groups are present in consistent proportions, representing 57% and 32% of total S_org_, respectively (both ± 3%, 1σ). Comparable mixtures of alkyl sulfides and thiophenes were generated in laboratory sulfurization experiments with functionalized OM and polysulfides^18,46^. A similar balance between these forms of reduced S_org_ was also observed in modern TOC- and S_org_-rich sediments from the Peru Margin (below 3 m sediment depth) and in shales from the Miocene Monterey Formation, suggesting that similar sulfurization processes may have operated during their deposition^16,41^. Rapid OM sulfurization reactions under a wide variety of conditions thus appear to generate a consistent blend of reduced S_org_ species, with notable contributions from alkyl sulfides.

Sulfurization processes during the formation of low-TOC rocks at Pont d’Issole generated S_org_ with a different redox character than the high-TOC rocks. In the lower part of the profile (7–9 m), reduced S_org_ in the limestone–marls is relatively rich in thiophenes, while other low-TOC rocks (20–20.5 m) are more alkyl sulfide-rich (Fig. 3). Despite their variable redox compositions, the low-TOC samples behave similarly in terms of OM δ^34^S values, S:C ratio, and concentration. The resolvable and stratigraphically coherent changes in the relative abundances of aromatic and aliphatic reduced S_org_ in low TOC-rocks have the potential to provide valuable information about the depositional environment, although data from other sites is clearly needed. For example, if alkyl sulfides primarily result from reactions with polysulfides rather than HS^−^, the ratio of thiophenes to alkyl sulfides in reduced S_org_ might reflect oxidant availability near the sedimentary chemocline.

Oxidized species (sulfonates and sulfate esters) account for a relatively large proportion of S_org_ in low-TOC samples (Fig. 3d), although their total abundance still increases with TOC from ≤0.6 μmol/g in limestones to as much as 2.8 μmol/g in TOC-rich shales (carbonate-free basis). Marine sulfonate sources are only beginning to be understood and may include both primary inputs from marine planktonic communities^47^ and alteration products from the oxidation of organic sulfides^48^. Relatively abundant sulfonates in specific low-TOC samples (e.g., 27% of total Sorg at 11 m) are suggestive of organic sulfide oxidation products, while the smaller proportion of sulfonates (8 ± 2%) in shales is more likely primary input from the marine biosphere. On the other hand, sulfate esters are not detected in TOC-rich shales and are most abundant in some post-OAE2 limestones. Sulfate esters are common in the connective tissue, polysaccharides, and excreta of animals, higher plants, and some algae^49^, and their presence may reflect a greater contribution of OM from the aerobic biosphere. Finally, although disulfides are thought to be common products of rapid sulfurization^50^, they represent relatively large proportions of S_org_ in a some low-TOC samples from Pont d’Issole. Disulfide-rich macromolecules can be generated experimentally in the presence of ammonia^51^, which suggests that disulfide abundance could indicate the availability of ammonia in the environment.

In sum, the two distinct depositional regimes of OM sulfurization in Pont d’Issole sediments appear to have left imprints on the redox character of S_org_. Under strongly O_2_-limited conditions, when sulfide, fresh OM, and abundant oxidants were available in sinking particles or surface sediments, rapid OM sulfurization produced abundant, strongly ^34^S-depleted S_org_ predominantly as alkyl sulfides. When the water column and sediment-water interface were oxic, a smaller amount of more degraded OM first encountered sulfide in deeper sediments, producing small amounts of sulfurized OM with a lower S:C ratio, a relatively ^34^S-enriched isotopic composition, and a more variable redox character that may record contributions from gradual sulfurization reactions, reoxidation events and/or the aerobic biosphere.

To understand how OM sulfurization processes fit into the evolving models for the onset and maintenance of OAE2, we need a mathematical framework for exploring the effects of sulfurization on OM preservation. At this point, however, we have few quantitative constraints on how rapid sulfurization might impact local carbon preservation under changing local redox conditions. What role did the activation of this preservation mechanism play in the nearly ten-fold change in OM concentrations between TOC-rich shales and limestones at Pont d’Issole?

**Fig. 4:**
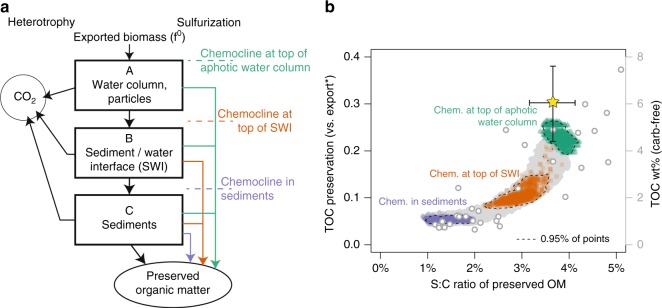
Sedimentary organic carbon cycle model. **a** Organic carbon fluxes in the model (see text). **b** Model results for the parameters in Table 1 and Supplementary Table 3 for sulfurization in all three boxes (chemocline at the top of the water column aphotic zone; green), boxes B and C (chemocline at the top of the sediment-water interface; orange), and c only (chemocline in the sediments; purple). Gray shading shows the effects of splitting boxes A or B into oxic and anoxic parts, representing a continuously moving chemocline. Cariaco parameters for box C export (“final” OM) and their fitting tolerances are shown as the yellow star. Pont d’Issole data in TOC wt% (carbonate-free basis, Supplementary Table 1) are shown on the right-hand axis as open gray circles

In an effort to answer this question, we developed a idealized mass balance for organic carbon flow through a sedimentary system equivalent to our best available modern analog, Cariaco Basin (Fig. 4), where OM sulfurizes in sinking particles as well as in underlying sediments^21,52–54^. To match the resolution of the Cariaco water column data, the model system is composed of three environments, representing: A) sinking particles in the water column below the photic zone, B) the sediment–water interface, and C) deeper sediments. In each box, organic carbon can be remineralized to CO_2_, sulfurized to “preserved” OM, or passed to the subsequent box. Primary biomass is divided into three different reactivity classes, as is typically called a “multi-G” model, with reactivities spanning previously inferred rate constants for Cariaco Basin (see Supplementary Note 3 and Supplementary Table 3) and relative abundances fit to Cariaco data^4,55^. We use the same reactivity vector for heterotrophy and sulfurization because similar functional groups are favorable for both reactions. Across all reactivity classes and environments, we assume that the rates of heterotrophy and sulfurization are saturating functions of electron acceptor and sulfide concentrations, respectively, and that all substrates except OM are replete. In the model, the rates of heterotrophy and sulfurization for each reactivity class are linear functions of OM concentration.

Rapid sulfurization occurs in particles and at the sediment-water interface, and gradual (kyr-scale) sulfurization occurs within the underlying sediments; this is in contrast to previous models for euxinic basins that included only gradual sulfurization in sediments^22^. Photic zone rapid sulfurization processes presumably do not occur in Cariaco Basin due to its 250-m–deep chemocline and are thus not represented in this calculation, which assumes a fixed export of total biomass OM below the photic zone. The combination of sulfurized OM from all boxes and remaining biomass exported from box C represents the total amount of organic carbon preserved.

With this simple mass balance exercise we can explore whether, in the absence of other changes, the effect of shifting the position of the chemocline on OM sulfurization is large enough to explain our observations. Importantly, this model does not aim to locate the chemocline mechanistically in the environment, but simply to explore the implications of changes in the position of the chemocline.

Potential ranges for sinking/burial times in each box are estimated based on observations from the literature, including particle settling rates^54^ and sediment accumulation rates from Cariaco^56^, as well as typical bioturbation/mixing depths for oxic conditions^57^. Model results are insensitive to realistic ranges of sinking/burial times, as shown in Supplementary Figure 3. In contrast, results are moderately sensitive to the assigned molar S:C ratio of rapidly sulfurized OM, which essentially represents the number of carbon atoms preserved for each S addition (Supplementary Figure 3). In Fig. 4, we assigned S:C ratios for gradual sulfurization, using the stoichiometry of individual organic compounds that are known to sulfurize over kyr (1–3%), and for rapid sulfurization, using observations from strongly sulfurized deposits and laboratory experiments (4–5%). These estimates ignore potential other factors that may have contributed to OM preservation in these example environments, like mineral protection^4^, which would cause the S:C ratios for rapid sulfurization to be underestimates of the effective carbon preservation per sulfur atom.

Using observed sulfur and carbon fluxes from Cariaco Basin and randomly picked values within our assigned ranges of sinking/burial times within each box and S:C ratios (Table 1), we solved 1000 times for a set of four unknowns: (1) the rate constant for sulfurization; (2) the rate constant for heterotrophy (both of which are scaled by the corresponding reactivity coefficient for each class of organic matter); (3) the initial relative abundance of organic matter in the second reactivity class; and (4) the initial relative abundance of organic matter in the third reactivity class (Supplementary Figure 2). With this set of solutions, we then conducted a series of experiments in which heterotrophy always occurs, but sulfurization is toggled on and off in boxes A and B to represent different positions of the chemocline in the environment. As described in detail in the Supplementary Information, we also tested two additional variants of this model. First, we subdivided Boxes A and B into well-mixed oxic and anoxic parts to illustrate the effects of moving the chemocline anywhere between the bottom of the photic zone (top of box A) and the bottom of the sediment-water interface (bottom of box B; gray shading on Fig. 4; see also Supplementary Figure 3 and Note 1). Second, we replaced the assumption that each box was well mixed with an assumption that organic matter concentration changes continuously within each environment as it moves in a strictly downward direction. The model predictions based on well mixed vs. continuous formulations differ only slightly (Supplementary Figure 4 and Note 2).

**Table 1:** Parameters used in organic carbon cycle model

Constraints from Cariaco Basin		Literature-derived value	Allowed 1*σ* range for fit
f0 (export production)	–>A	100%	
C flux (vs. f0)	A–>B	51%	15%
	B–>C	38%	5%
	C–>final	30%	8%
OM S:C (mol/mol)	A–>B	Poorly constrained	
	B–>C	2.0%	0.5%
	C–>final	3.7%	0.5%
Assigned parameter ranges		min	max
Turnover time	A	0.5 weeks	3 weeks
	B	2 years	500 years
	C	2000 years	20,000 years
Effective S:C ratio	Biomass	0%	1%
	Rapid sulf. (A + B)	4%	5%
	Gradual sulf. (C)	1%	3%

The difference in predicted OM burial between different chemocline positions in this mass balance exercise approximates the contribution of rapid OM sulfurization to TOC burial in a Cariaco Basin-like environment. Relative to the control case with sulfurization in all boxes, modeled TOC preservation is an average of 55% lower when the chemocline is near the sediment-water interface and 77% lower when the chemocline is in deeper sediments (Fig. 4). This scale of change in TOC burial for different redox conditions is comparable to the difference we observe between shales and limestones at Pont d’Issole, which averages 78% on a carbonate-free basis (or 87% on a whole-rock basis, Supplementary Table 2). Using reasonable ranges of environmental parameters and Cariaco Basin as an analog, we can thus reproduce the relationship between S:C ratio and TOC burial that we observe in Pont d’Issole sediments by varying only whether boxes A and B are reducing enough to support OM sulfurization. By this mechanism, modest changes in the vertical position of the chemocline near the sediment-water interface have the ability to dramatically affect the age, reactivity, and functional group density of the OM available for sulfurization, driving dynamic changes in local carbon burial at Pont d’Issole and other sites with fluctuating redox conditions. We conclude that the observed changes in OM burial across this section are unlikely to reflect ten-fold swings in original primary productivity, but instead largely reflect the effects of changing local redox state on the rate and location of OM sulfurization.

Changes in the intensity of OM sulfurization at Pont d’Issole occurred in the context of myriad global changes before and during OAE2. Productivity was almost certainly enhanced to some degree due to sea level change, inputs of nutrients associated with large igneous province volcanism and enhanced weathering^58^, and/or the low phosphorus retention capacity of anoxic sediments^59,60^, which should have increased the supply of nutrients for primary producers. Circulation patterns in the ocean also changed in response to changing climate^35^. In this context, OM sulfurization could have a critical role in facilitating globally elevated OM burial, amplifying the impacts of modest increases in primary productivity that result in a shallowing of the chemocline into the realm of rapid OM sulfurization – the sediment–water interface or water column.

This threshold, above which sulfide is available to react with abundant, relatively labile OM, has major implications for the global carbon cycle. Local environments that cross that threshold are likely to have an outsize impact on global TOC fluxes, consistent with recent global budgets focusing global OM burial in localized, shallow environments^2^. The activation of rapid OM sulfurization due to chemocline shoaling could also help explain how a gradual forcing like sea level or climate change might lead to a large, geologically sudden change in OM burial at the onset of OAE2. In the 150,000 years or so leading up to OAE2, evidence from transition metals suggests that large areas of the Tethyan Ocean became sub-oxic in response to higher productivity and/or changing climate^61^. These areas would have been poised near the critical threshold for rapid OM sulfurization and therefore could have buried large amounts of OM in response to a relatively small increase in effective O_2_ limitation, driving a large, sudden OM burial flux and the sharp δ^13^C signal at the onset of OAE2. Because the OM preserved by this sulfurization is S-rich, S_org_ burial is also likely to be a major sink of ^34^S-depleted sulfur in the global sulfur cycle, influencing the δ^34^S value of seawater sulfate alongside pyrite.

## Methods

### Elemental and isotopic analysis

Samples were prepared and analyzed for C and S isotopic compositions at Washington University in St. Louis. Rocks were first powdered in a ceramic shatterbox and then washed four times in deionized water and lyophilized. Solvent-extractable organics (bitumen) and elemental S were microwave–extracted from the rock powders into 9:1 dichloromethane (DCM):methanol (at 60° for 20 min, twice; MARS 6, CEM Corp.). This solvent-extractable S is excluded from discussion in this paper; we use “S_org_” to refer to the residual organic S (i.e., that in the operationally defined kerogen pool), which represents the vast majority (96.6–99.3%) of total sedimentary organic S (Supplementary Table 1).

Acid-volatile and chromium-reducible sediment fractions were then collected in series using standard methods. Sediments were extracted with 6N HCl (180°, 4 h), which also fully removed carbonates, and then with HCl and CrCl_2_ (180°, 4 h) to reduce pyrite (FeS_2_) to sulfide gas^62,63^. In both cases, H_2_S was trapped in 0.5 M zinc acetate solution as ZnS and rinsed in ultra-pure water. For quantification and isotopic analysis, ZnS was converted to sulfate using hydrogen peroxide (two rounds of 2–5 ml 30% H_2_O_2_, heated to 70 °C for 24 h and dried on a hot plate in Teflon vials). Residual sediments were washed with 0.5N HCl and extensively with ultra-pure water and dried. Remaining carbon, nitrogen, and sulfur in this material are presumed to represent predominantly kerogen. Carbonate contents were estimated from sediment mass loss.

Sulfate concentrations for the solvent-extractable, AVS, and CRS fractions were measured using a Metrohm 881 ion chromatograph on a Metrosep A Supp 7 150 × 4.0 mm anion column. Aliquots of sufficiently large sulfate samples (>1 μmol S) were precipitated as BaSO_4_ with excess BaCl_2_ in ultra-pure water, rinsed, and dried for subsequent isotopic analysis. BaSO_4_ and residual sediment samples were weighed into tin capsules with V_2_O_5_ and analyzed by combustion EA-IRMS (Costech 4010 EA + Thermo Delta V Plus, configured for S). Results are reported as δ^34^S values on the V-PDB scale based on regular analyses of IAEA-S3 and in-house standards. Organic carbon and nitrogen concentrations and δ^13^C values for residual sediment were analyzed by EA-IRMS (Thermo Flash 2000 EA with zero-blank autosampler + Delta V Plus, configured for C and N). δ^13^C values are reported relative to V-CDT based on standards USGS-40 and 41. Concentrations of S, C, and N in sediments are quantified based on thermal conductivity detector response. Long-term analytical uncertainties are approximately ± 0.5‰ for δ^34^S and ± 0.2‰ for δ^13^C.

### XAS analysis

XAS was conducted at beamline 14-3 at the Stanford Synchrotron Radiation Lightsource (SSRL) at the SLAC National Accelerator Laboratory. Beamline 14–3 is a bending magnet workstation operating between 2100 and 5000 eV with a flux of 2 × 10^10^ photons/second. In this study, the beam size was set to 500 × 1000 μm and x-ray energy was calibrated to the sodium thiosulfate (Na_2_S_2_O_3_) pre-edge peak at 2472.02 eV. Sequentially extracted rock powders from S-isotope and C-isotope analysis were adhered onto Saint Gobain M60 S-free polyester tape and covered in 5-μm-thick SPEX 3520 polypropylene XRF film. The sample chamber was filled with He. Multiple spectra (3–5 replicates) were collected between 2460 and 2540 eV. Spectrum averaging, background removal, and pre-edge height normalization were conducted in the SIXPACK software package^64^ using a K-edge E^0^ of 2470 and pre-edge and post-edge normalization ranges of –20 to –7 and 26 to 70 eV, respectively. Energies from January and April spectra were adjusted by +0.19 and –0.11 eV, respectively, to align standards and replicate sample results prior to fitting. Spectra were fitted to a set of in-house standards using a least-squares fitting routine, also in SIXPACK.

### Code availability

Model code in R is available from M.R.R. or H.-L.O.M. on request.

### Data availability

All of the data described in this manuscript are included in this published article and its supplementary information files. Pre-processed XAS spectra are available upon request.

## Electronic supplementary material

Supplementary Information
