# Supplementary material for: Organic carbon burial during OAE2 driven by changes in the locus of organic matter sulfurization
Source: Nat Commun. 2018 Aug 24;9:3409. doi: 10.1038/s41467-018-05943-6 (PMC6109118; doi:10.1038/s41467-018-05943-6)
Supplement: Supplementary file 1 — Supplementary Information [file 41467_2018_5943_MOESM1_ESM.pdf]

Supplementary Information

Raven et al.

Organic carbon burial during OAE2 driven by changes in the locus of organic matter sulfurization

Supplementary Note 1 – Model derivation and reaction rates

We model the water / sediment column as consisting of three distinct environments, A, B and C, which refer approximately to the water column, the sediment-water interface, and buried sediments, respectively. Organic matter moves sequentially from the shallowest to deepest boxes. Export from each box is the sum of organic matter that sulfurized in each box, plus the sum of organic matter still intact. The final pool of "preserved" OM includes the OM sulfurized in each box plus any residual OM that has not been remineralized by the end of the box C window.

At all depths, the concentration of primary organic carbon is a 1D array of the form:

$$\boldsymbol{G} = \begin{pmatrix} \boldsymbol{G_1} \\ \boldsymbol{G_2} \\ \boldsymbol{G_3} \end{pmatrix}, \qquad [1]$$

where 1, 2 and 3 refer to grades of organic matter divided on the basis of relative reactivity, and G is a function of depth, z, and time, t. Relative reactivity of organic matter fractions is given by a reactivity parameter that we here call 'juiciness', J:

$$\boldsymbol{J} = \begin{pmatrix} J_1 = 1.0 \\ J_2 = 0.01 \\ J_3 = 0.0001 \end{pmatrix} \qquad [2]$$

The assigned four-order-of-magnitude difference between J1 and J3 is designed to approximate the observed timescales of reactivity for rapid and gradual sulfurization in Cariaco Basin; Werne et al. calculated a first-order rate constant for gradual sulfurization of one compound of 2 x 10^{-4}/yr, and we estimate apparent rates in particles to be between 2 – 20 /yr based on published Cariaco Basin fluxes [1,2]. Sensitivity to this selection for the range of J values is minor (Supplementary Figures 3 and 4).

In our model set-up, the chemocline can move from the sea surface (top of environment A) to the bottom of the sediment water interface (bottom of environment B). In the model, the position of the chemocline simply determines whether or not sulfurization occurs: above the chemocline, where it is oxic, S = 0. Our model therefore consists of 5 boxes: A_{Upper}(oxic), A_{Lower}(anoxic) (collectively, box A), B_{Upper}(oxic), B_{Lower}(anoxic) (collectively, box B), and C. When the chemocline is between environments, only 3 of the boxes are active, and when the chemocline divides an environment with an oxic upper part and an anoxic lower part, the model consists of 4 active boxes.

Rates of both sulfurization and heterotrophy are first order for OM only (i.e., sulfide and electron acceptors are treated as non-limiting), and rate constants scale proportionally to J:

$$\boldsymbol{S} = s\boldsymbol{J} \text{ and } \boldsymbol{H} = h\boldsymbol{J}, \qquad [3]$$

where s and h are scaling constants. Implicitly, this treats generalized organic functional groups as similarly reactive toward both abiotic sulfurization as toward fermentative enzymes. The rate of heterotrophy in each pool is then:

$$\boldsymbol{Gh_A} = \boldsymbol{G_A}h\boldsymbol{J}$$
$$\boldsymbol{Gh_B} = \boldsymbol{G_B}h\boldsymbol{J}$$
$$\boldsymbol{Gh_C} = \boldsymbol{G_C}h\boldsymbol{J}. \qquad [4]$$

Rate of sulfurization, in units of carbon sulfurized, in any pool is then:

$$\boldsymbol{Gs_A} = \boldsymbol{G_A} s \boldsymbol{J} C_A$$

$$\boldsymbol{Gs_B} = \boldsymbol{G_B} s \boldsymbol{J} C_B$$

$$\boldsymbol{Gs_C} = \boldsymbol{G_C} s \boldsymbol{J} C_C, \qquad [5]$$

and where C represents the position of the chemocline, by taking a value of 0 (no sulfide present), or 1 (non-limiting sulfide present). The flux from box A to B, B to C, and from C to K (indefinite preservation) is given by a prescribed (or constrained) set of export rate constants (1/residence time in box) such that:

$$\boldsymbol{F_{AB}} = f_A \boldsymbol{G_A}$$

$$\boldsymbol{F_{BC}} = f_B \boldsymbol{G_B}$$

$$\boldsymbol{F_{CK}} = f_C \boldsymbol{G_C} \qquad [6]$$

In each box the input is from above and the outputs are heterotrophy, sulfurization or sinking intact. The mass balance for organic C in each box is a balance between sulfurization, heterotrophy, and flux out to the subsequent box, in steady state:

$$\frac{d\boldsymbol{G_A}}{dt} = 0 = \boldsymbol{F_{0A}} - \boldsymbol{Gh_A} - \boldsymbol{Gs_A} - \boldsymbol{F_{AB}} = \boldsymbol{F_{0A}} - \boldsymbol{G_A}(f_A + \boldsymbol{H} + \boldsymbol{S}C_A)$$

$$\frac{d\boldsymbol{G_B}}{dt} = 0 = \boldsymbol{F_{AB}} - \boldsymbol{Gh_B} - \boldsymbol{Gs_B} - \boldsymbol{F_{BC}} = \boldsymbol{G_A} f_A - \boldsymbol{G_B}(f_B + \boldsymbol{H} + \boldsymbol{S}C_B)$$

$$\frac{d\boldsymbol{G_C}}{dt} = 0 = \boldsymbol{F_{BC}} - \boldsymbol{Gh_C} - \boldsymbol{Gs_C} - \boldsymbol{F_{CK}} = \boldsymbol{G_B} f_B - \boldsymbol{G_C}(f_C + \boldsymbol{H} + \boldsymbol{S}C_C) \qquad [7]$$

where C represents the position of the chemocline, prescribed as either 0 (no sulfide present) or 1 (non-limiting sulfide present). Total sulfurization in each pool is the dot

product of G and S. The final pool of "preserved" OM includes the OM sulfurized in each box plus any residual OM that has not been remineralized by the end of the box C window. Assuming steady state and rearranging the above differential equations for G in each box gives:

$$\frac{\boldsymbol{F_{0A}}}{(f_A\boldsymbol{I} + \boldsymbol{H} + \boldsymbol{S}C_A)} = \boldsymbol{G_A}$$

$$\frac{\boldsymbol{F_{0A}}f_A\boldsymbol{I}}{(f_B\boldsymbol{I} + \boldsymbol{H} + \boldsymbol{S}C_B)(f_A\boldsymbol{I} + \boldsymbol{H} + \boldsymbol{S}C_A)} = \boldsymbol{G_B}$$

$$\frac{\boldsymbol{F_{0A}}f_A\boldsymbol{I}f_B\boldsymbol{I}}{(f_C\boldsymbol{I}+\boldsymbol{H}+\boldsymbol{S}C_C)(f_B\boldsymbol{I}+\boldsymbol{H}+\boldsymbol{S}C_B)(f_A\boldsymbol{I}+\boldsymbol{H}+\boldsymbol{S}C_A)} = \boldsymbol{G_C} \quad [8]$$

The total rate of sulfurization in each pool is the dot product of G and S:

$$|\boldsymbol{Gs_A}| = \boldsymbol{G_A} \cdot \boldsymbol{S}C_A = G_{1A}S_1C_A + G_{2A}S_2C_A + G_{3A}S_3C_A$$

$$|\boldsymbol{Gs_B}| = \boldsymbol{G_B} \cdot \boldsymbol{S}C_B = G_{1B}S_1C_B + G_{2B}S_2C_B + G_{3B}S_3C_B$$

$$|\boldsymbol{Gs_C}| = \boldsymbol{G_C} \cdot \boldsymbol{S}C_C = G_{1C}S_1C_C + G_{2C}S_2C_C + G_{3C}S_3C_C. \quad [9]$$

Parameters were either prescribed or allowed to vary and be fitted to a range of realistic observable outputs (Supplementary Table 3). A range of realistic values were prescribed for S:C ratios organic matter sulfurized in each box (assumed to be constant throughout each box), and for the thickness of each box and the sinking velocity within each box. The parameters that were allowed to vary are: h and s (the rate constants for the rate of heterotrophy and sulfurization respectively), and G_{02} and G_{03} (the relative abundance of organic matter that correspond to 'juiciness' values J_2 and J_3 respectively. The outputs used to constrain these parameters were from Cariaco basin (see below); the S:C ratio of

organic matter exported from box B and from box C, and the total organic carbon preservation efficiency at the bottom of box A, box B and box C.

We fitted the unknown parameters for an entirely anoxic water column. Firstly, we randomly sampled the input parameters and the constraining parameters within their prescribed ranges (across a uniform distribution) a large number of times. Secondly, with each randomly selected parameter set, we fitted the unknown parameters using a locally-biased dividing-rectangles algorithm (DIRECT-L) implemented in the nloptr package for R (1. J. M. Gablonsky and C. T. Kelley, "A locally-biased form of the DIRECT algorithm," J. Global Optimization, vol. 21 (1), p. 27-37 (2001) ; 2. Steven G. Johnson, The NLopt nonlinear-optimization package, http://ab-initio.mit.edu/nlopt). Using the constrained values for each pseudo-replicate dataset, we then explored the effect of turning on and off sulfurization in each box (and with a variable chemocline).

Supplementary Note 2 – Alternative model derivation, continuous G formulation

As an alternative formulation, we drop the assumption that all boxes are all well mixed, and instead assume that organic matter moves in a strictly downward direction, reacting continuously as it passes through each box. All other aspects of fitting to Cariaco data and subsequent experiments remain unchanged.

G as a function of time, t, and depth, z, is given by:

$$\frac{\partial G}{\partial t} = -(H + S)G - v_z \frac{\partial G}{\partial z}, \qquad [10]$$

where v_z is the sinking / burial velocity of organic matter at depth, z, and movement of organic matter is strictly in the direction of increasing z. At steady state, the time derivative of G is zero, so we can derive an expression for G as a function of z:

$$\int \frac{1}{G} \partial G = -\int \frac{(H+S)}{v_z} \partial z, \qquad [11]$$

$$\ln G = -\frac{(H+S)}{v_z} z + const., \qquad [12]$$

$$\mathrm{G} = G_0 e^{-\frac{(H+S)}{v_z} z}, \qquad [13]$$

where G0 is the value of G at z = 0.

Like the mass balance calculation with well mixed boxes in Supplementary Note 1, the chemocline can move from the sea surface (top of environment A) to the bottom of the sediment water interface (bottom of environment B). and our model therefore consists of 5 boxes: When the chemocline is between environments, only 3 of the boxes are active, and when the chemocline divides an environment with an oxic upper part and an anoxic lower part, the model consists of 4 active boxes. If it is within an environment, the position of the chemocline determines the relative thickness of each box. We assume v to be constant throughout each of the environments. Within box i the total rate of heterotrophy is therefore given by the dot product of H and the integral of G over the depth of the box:

$$\mathrm{H} \cdot \int_0^{Z_i} \mathrm{G} = \mathrm{H} \cdot G_0 \frac{v_i}{(H+S)} \left(1 - e^{-\frac{(H+S)}{v_i} Z_i}\right) , \qquad [14]$$

where Zi and vi are respectively the thickness of box i and velocity of sinking within box, I, and G0 is the concentration at the top of the box. Similarly the total rate of sulfurization in box, i, is given by:

$$S \cdot \int_0^{Z_i} G = S \cdot G_0 \frac{v_i}{(H+S)} \left(1 - e^{-\frac{(H+S)}{v_i} Z_i}\right) . \qquad [15]$$

The flux of organic matter from one box to the next is given by the product of the sinking velocity and the concentration of the remaining organic matter:

$$F_{iout} = v_i G(Z_i) = G_0 e^{-\frac{(H+S)}{v_z} Z_i}. \qquad [16]$$

NB: The concentration of organic matter at the top of the subjacent box, is not equal to the concentration at the bottom of the superjacent box, and must be scaled by the ratio of velocities. Final preserved organic matter is the sum of organic matter sulfurized in each box and any surviving organic matter exported from box C.

<u>Supplementary Note 3 – Assigned parameters and sensitivity tests</u>

The model is most sensitive to the prescribed ratio of S:C for sulfurized OM. Essentially, this ratio represents the number of carbon atoms preserved for each added S atom. Common organosulfur compounds that have been identified in both experimental systems and ancient sediments (e.g., C_{20} –C_{30} isoprenoid thiophenes) have effective S:C molar ratios between 3.3 and 5% [3,4]. Rapidly sulfurized dissolved OM from porewater is reported at a similar ratio of 3.7%, although some experimental products reached as much as 10% [5]. Strongly sulfurized bulk OM has an S:C ratio of ~6% in the Kimmeridge Clay [6], ~7.0% in OAE2 Demerara Rise sediments [7]. For this study, we explored a range of S:C values between 4 and 6%, similar to both apparent "full sulfurization" of many TOC-rich

deposits and equivalent to the elemental formulae of common identified sulfurized lipid structures.

A chemically reasonable upper bound for S:C ratios is ~12%, which is equivalent to the average structure of the small alkyl(benzo)thiophenes commonly released from kerogens by pyrolysis [8,9]. Although organic polysulfides could yield higher ratios, they are unlikely to be stable on geologic timescales and we do not observe them in Pont d'Issole rocks by XAS. If effective S:C ratios at Pont d'Issole were higher than 6.0 (but still presumably less than ~12), our model would overestimate the contribution of rapid sulfurization to C preservation.

For gradual (sedimentary) sulfurization, we selected S:C ratios between 1% and 3%. We assign lower S:C ratios for gradual sulfurization because we expect organic molecules in the slowly-reactive pool (e.g., algaenan) to be larger and less densely populated with functional groups than highly reactive OM (e.g., sugars).

The model was made to output parameters that could be fitted to data from Cariaco basin by searching parameter space. These parameters were the S:C ratio of matter exported from box B, and from box C and the total relative export flux of organic carbon from box A, B and C. The misfit function was constructed as the sum of squared differences between these 5 parameters output by the model and the data from Cariaco, with the importance of each parameter weighted by the inverse of the associated uncertainty. The

fitting routine was undertaken in R using the DIRECT-L algorithm in the Nlopr package [10].

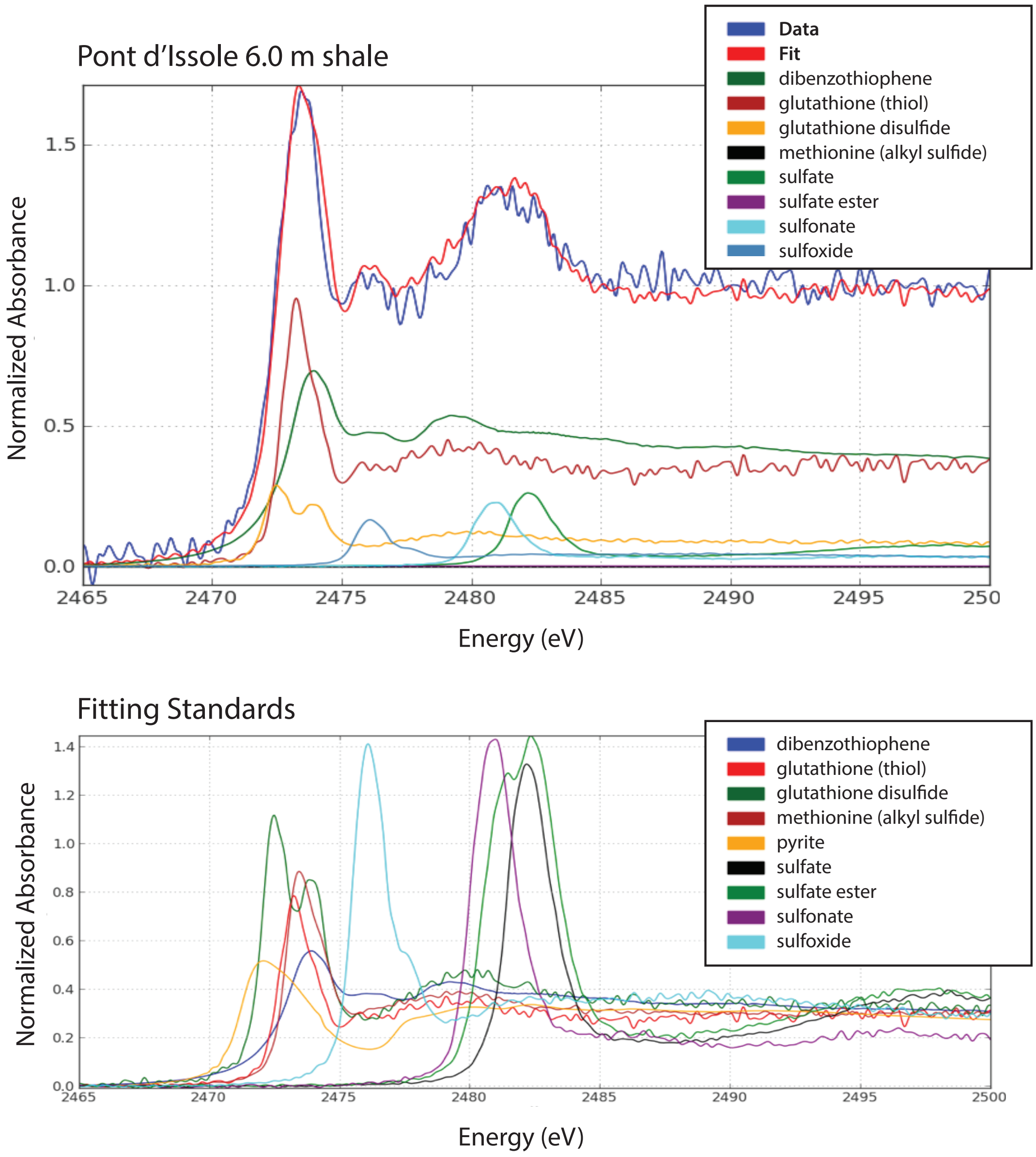

Supplementary Figure 1 – Example XAS spectra for Pont d'Issole. The upper panel shows the spectrum of organic S from 6.0 m and the lower panel shows a compilation of standards used for fitting data in SIXPACK. [11]

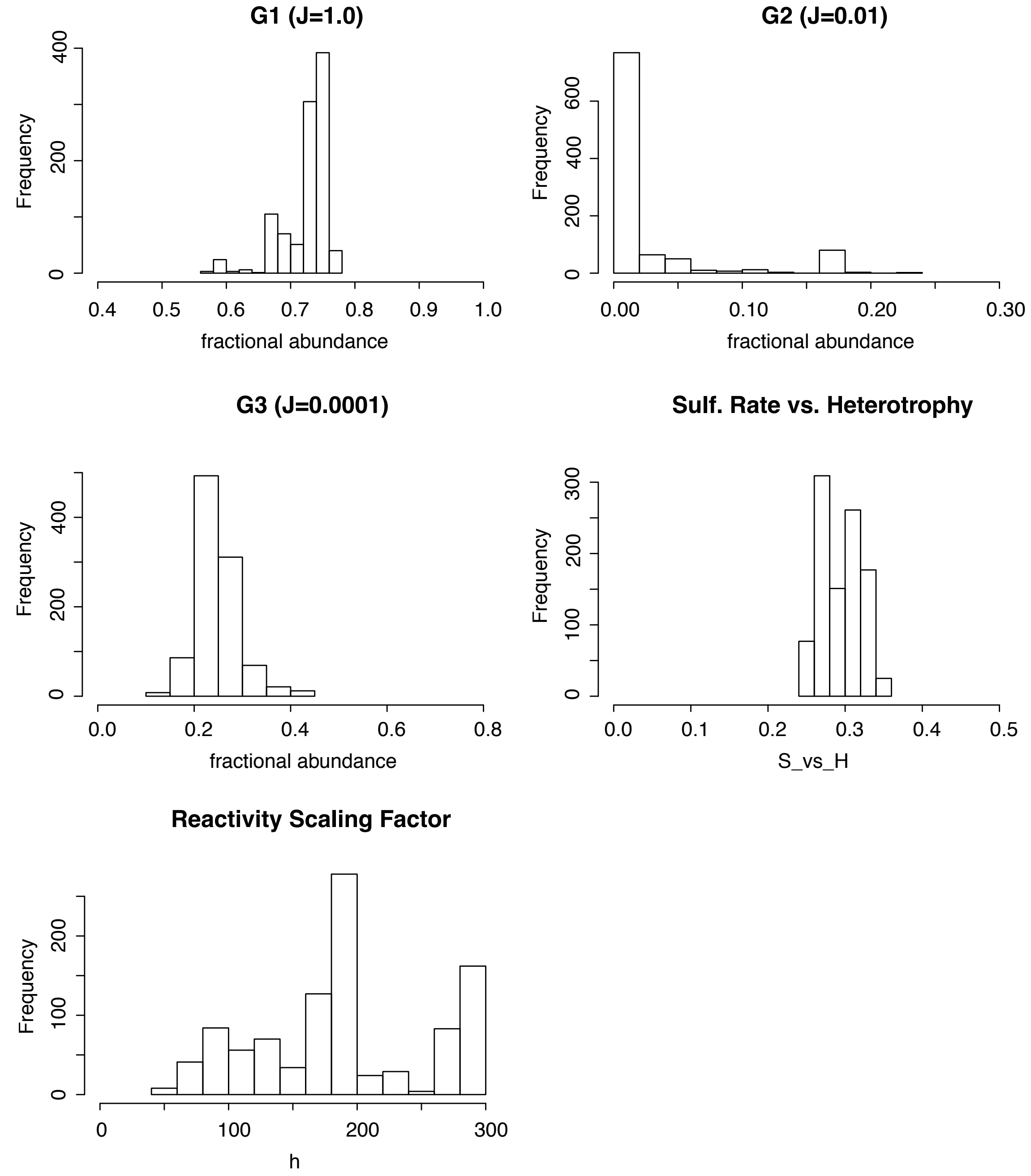

Supplementary Figure 2 – Distribution of model solutions fitted to Cariaco Basin data. Inputs were randomly selected from assigned ranges in reasonable turnover times

and effective S:C ratios and used to generate each cloud of data in Fig. 4 (10,000 realizations). G1, G2, and G3 refer to the relative abundances of fractions of OM with different reactivities (J=1.0, 0.01, and 0.0001, respectively). These fractions and the ratio of sulfurization to heterotrophy are reproducibly constrained by the selected ranges of input parameters (Table 1). The model assumes a single S_vs_H ratio for all three boxes. The ‘reactivity scaling factor’ essentially turns OM reactivity up or down, matching the assigned ‘juiciness’ values to the total heterotrophy and sulfurization implied over the characteristic timescale of each box.

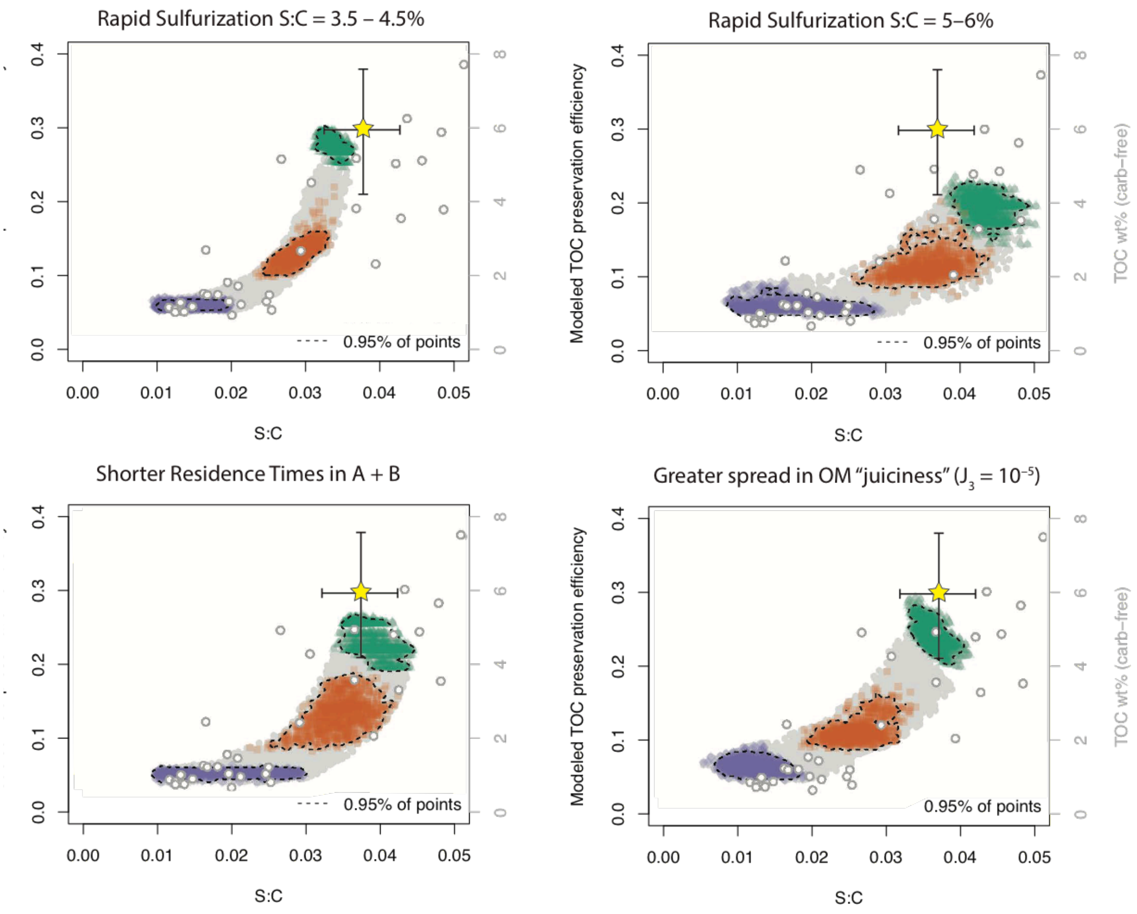

Supplementary Figure 3 – Model sensitivity to assigned parameters. All parameters are kept constant except those noted; see Supplementary Table 3 for details. Purple, orange, and green points represent results for experiments with sulfurization in one, two, or three boxes, respectively, and grey shading represents the effects of dividing box A or B into oxic and anoxic parts. The yellow star represents Cariaco Basin data with fitting tolerances. Open circles are Pont d'Issole data on the right-hand y-axis.

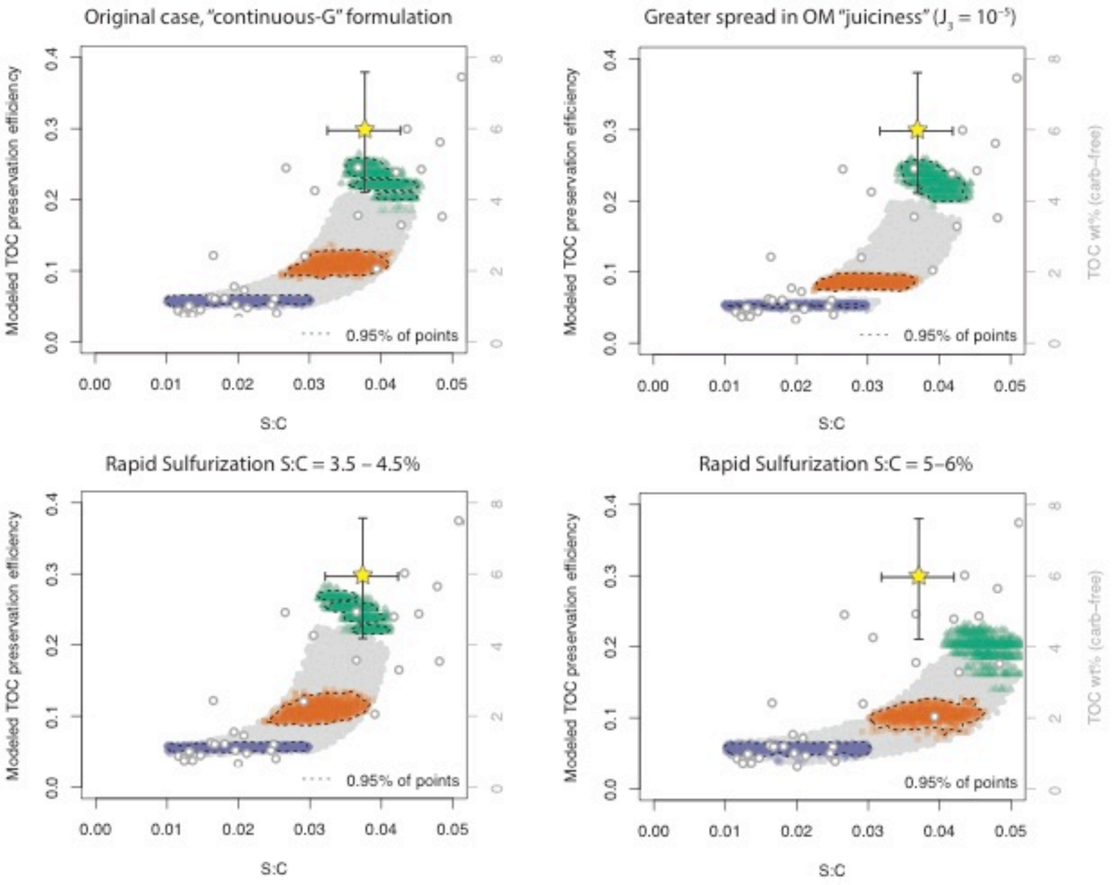

Supplementary Figure 4 – Alternative "continuous-G" model sensitivity to assigned parameters. All parameters are kept constant except those noted; see Supplementary Table 3 for details. Purple, orange, and green points represent results for experiments with sulfurization in one, two, or three boxes, respectively, and grey shading represents

the effects of dividing box A or B into oxic and anoxic parts. The yellow star represents Cariaco Basin data with fitting tolerances. Open circles are Pont d'Issole data on the right-hand y-axis.

Supplementary Table 1: geochemical data for Pont d'Issole rocks

Sample name	distance (m)	rock type	carbonate	OM $\delta^{13}C$	[TOC] carb-free	[TOC] whole-rock	[S-org]	[S-pyrite]	OM $\delta^{34}S$	pyrite $\delta^{34}S$	py-org $\Delta\delta^{34}S$	[Fe-tot] carb-free	OM S:C	OM N:C
			wt%	‰ (VCDT)	wt%	wt%	µmol/g	µmol/g	‰ (VPDB)	‰ (VPDB)	‰	µmol/g	mol:mol	mol:mol
PdIc-0.0	0	limestone	89%	-26.0	0.59	0.07								
PdIc-0.5	0.5	limestone	89%	-25.3	0.57	0.06								
PdIc-1.8	1.8	limestone	88%	-25.4	0.58	0.07								
PdIc-4.0	4	shale	65%	-26.9	0.10	0.03		65.0		-9.4		940		0.042
PdIc-4.5	4.5	shale	62%	-27.5	3.52	1.34	20.1	45.2	-22.8	-32.4	9.6	746	0.048	0.043
PdIc-5.0	5	shale	62%	-26.4	0.86	0.33	1.2	42.5	-15.2	-30.2	15.0	807	0.012	0.050
PdIc-5.5	5.5	shale	38%	-26.7	3.55	2.19	25.1	54.3	-23.2	-20.2	-3.0	715	0.037	0.038
PdIc-6.0*	6	shale	57%	-26.3	2.40	1.04	9.5	78.5	-19.5	-40.6	20.8	937	0.029	0.036
PdIc-6.5	6.5	shale	60%	-25.5	4.85	1.94	27.5	49.9	-27.8	-36.4	8.6	826	0.045	0.034
PdIc-7.0	7	shale	74%	-25.9	1.20	0.32	2.5	13.2	-19.1	-37.6	18.5	813	0.025	0.037
PdIc-7.2	7.2	limestone	79%	-25.7	1.02	0.21	1.6	37.9	-18.4	-39.8	21.4	983	0.025	0.044
PdIc-7.7	7.7	shale	80%	-25.2	0.98	0.20	0.8	1.0	-17.3	-23.0	5.8	998	0.013	0.035
PdIc-8.0	8	limestone	80%	-23.3	0.39	0.08								
PdIc-8.42	8.42	shale	74%	-23.5	0.83	0.22								
PdIc-8.64	8.64	limestone	77%	-23.3	0.34	0.08								
PdIc-8.78	8.78	shale	76%	-25.8	0.81	0.19	0.7	0.8	-13.6	-20.1	6.5	900	0.012	0.039
PdIc-8.9	8.9	limestone	75%	-23.6	0.79	0.20						899		
PdIc-9.0	9	shale	76%	-26.0	0.99	0.23	1.0	0.5	-16.9			1139	0.013	0.033
PdIc-9.15	9.15	limestone	78%	-24.1	0.36	0.08						838		
PdIc-9.25	9.25	shale	79%	-25.1	0.67	0.14		0.7				815		0.042
PdIc-9.6	9.6	shale	70%	-24.9	0.76	0.23	0.9	0.6	-12.5				0.013	0.038
PdIc-9.95	9.95	shale	73%	-25.5	0.73	0.20	0.8	2.5	-15.9	-33.3	17.4		0.012	0.036
PdIc-11.0	11	shale	64%	-25.1	0.79	0.28	2.2	21.7	-21.9	-33.3	11.4	733	0.025	0.036
PdIc-11.5	11.5	shale	48%	-24.4	2.04	1.07	13.0		-23.6	-37.9	14.3	791	0.039	0.037
PdIc-12.0	12	shale	46%	-24.4	3.28	1.77	23.5	56.9	-29.7	-37.6	7.9	635	0.043	0.035
PdIc-12.5	12.5	shale	60%	-24.5	4.91	1.94	22.2	10.7	-30.6	-35.2	4.5	620	0.037	0.032
PdIc-13.0*	13	shale	60%	-24.3	1.54	0.61	3.7		-24.5	-32.8	8.3	725	0.019	0.036
PdIc-13.5	13.5	shale	68%	-25.3	0.65	0.21	1.3	1.2	-15.6	-19.5	3.9	825	0.020	0.045
PdIc-14.0	14	shale	59%	-24.4	7.46	3.05	48.5	59.5	-29.3	-27.8	0.0	709	0.051	0.033
PdIc-14.5	14.5	shale	56%	-24.7	4.77	2.12	27.7	59.1	-30.4	-32.5	3.2	565	0.042	0.033
PdIc-15.0	15	shale	61%	-24.2	5.62	2.17	32.5	61.8	-30.4	-26.5	-3.4	697	0.048	0.031
PdIc-15.5	15.5	shale	65%	-24.5	5.99	2.10	28.4	39.5	-30.1	-28.5	-5.7	634	0.043	0.029
PdIc-16.1	16.1	limestone	83%	-24.3	1.02	0.17	1.0	0.7	-18.3			783	0.020	0.024
PdIc-16.37	16.37	shale	74%	-24.8	1.44	0.38	2.4	24.9	-16.9	-9.0	-7.9	721	0.021	0.035
PdIc-17.1	17.1	shale	73%	-24.5	4.25	1.16	11.1	24.3	-21.7	-33.8	12.1	701	0.031	0.030
PdIc-18.0	18	shale	76%	-25.0	1.24	0.30	1.5	0.4	-13.6			820	0.016	0.034
PdIc-18.9	18.9	shale	73%	-25.8	1.20	0.32	1.7	32.5	-12.3	-30.7	18.4	753	0.017	0.035
PdIc-19.6	19.6	limestone	80%	-26.0	0.88	0.17	0.8	18.4	-10.9	-25.1	14.2	800	0.015	0.040
PdIc-20.0	20	limestone	84%	-27.6	1.21	0.19	1.1	26.9	-16.1	-24.8	8.7	736	0.018	0.031
PdIc-20.47*	20.47	shale	76%	-25.6	2.42	0.59	3.0	25.8	-11.8	-0.9	-10.9	706	0.017	0.027
PdIc-20.82	20.82	limestone	81%	-24.8	1.05	0.20								
PdIc-22.25	22.25	shale	81%	-26.3	0.94	0.18	1.2	33.9	-13.8	-24.9	11.1	801	0.021	0.039
PdIc-22.6	22.6	shale	78%	-26.3	0.74	0.16	0.7	23.3	-7.2	-11.4	4.2	712	0.014	0.044
PdIc-23.5	23.5	shale	76%	-25.5	4.89	1.17	9.7	36.0	-14.4	-36.2	21.8	614	0.027	0.028
PdIc-24.3	24.3	shale	87%	-26.0	1.09	0.14		0.7				614		0.029

* = average values for replicates

Supplementary Table 2: x-ray absorption spectroscopy results for Pont d'Issole kerogens

	Thiophene		Thiol		Disulfide		Alkyl Sulfide		Pyrite		Sulfate		Sulfate Ester		Sulfonate		Sulfoxide		Fit Quality Parameters			
height (m)	rel. conc	1σ	rel. conc	1σ	rel. conc	1σ	rel. conc	1σ	rel. conc	1σ	rel. conc	1σ	rel. conc	1σ	rel. conc	1σ	rel. conc	1σ	Chi-Sq	Red. Chi-Sq	R-val	Comp Sum
4.5	0.321	0.015	nd		0.074	0.005	0.506	0.010	0.010		nd		0.011	0.002	0.028	0.002	0.087	0.004	2.192	0.003	0.003	1.026
5.5	0.223	0.018	nd		0.108	0.006	0.567		nd		nd		0.006	0.002	0.028	0.003	0.100	0.004	2.829	0.004	0.003	1.031
6	0.703	0.014	0.044	0.017	nd		0.162	0.014	nd		0.017	0.002	nd		0.031	0.002	0.030	0.003	1.706	0.002	0.003	0.987
	0.505	0.013	0.135	0.015	0.087	0.005	0.155	0.013	nd		0.001	0.007	0.036	0.007	0.014	0.004	0.038	0.003	1.375	0.002	0.002	0.971
6.5	0.316	0.015	nd		0.060	0.005	0.534	0.010	nd		nd		0.011	0.002	0.016	0.002	0.087	0.003	2.024	0.003	0.003	1.023
7	0.509	0.021	nd		0.112	0.007	0.237	0.014	nd		nd		0.054	0.003	0.005	0.003	0.060	0.005	4.205	0.006	0.006	0.976
7.2	0.856	0.026	nd		nd		0.009	0.018	nd		nd		0.033	0.004	0.009	0.005	0.014	0.006	7.653	0.011	0.014	0.921
9	0.769	0.034	nd		0.097	0.011	0.017	0.023	nd		nd		0.027	0.005	0.035	0.005	0.036	0.008	11.048	0.016	0.016	0.981
11	0.945	0.056	nd		nd		nd		nd		0.035	0.008	nd		0.071	0.007	nd		29.266	0.053	0.050	1.051
11.5	0.410	0.014	nd		0.068	0.005	0.421	0.010	nd		nd		0.018	0.002	0.035	0.002	0.072	0.003	1.975	0.003	0.003	1.025
12	0.540	0.014	nd		0.034	0.005	0.315	0.009	nd		0.011	0.002	nd		0.032	0.002	0.053	0.003	1.859	0.003	0.003	0.985
12.5	0.452	0.013	nd		0.055	0.004	0.396	0.009	nd		nd		0.010	0.002	0.024	0.002	0.077	0.003	1.692	0.002	0.002	1.015
13	0.307	0.029	nd		0.052	0.009	0.397	0.018	nd		nd		0.066	0.015	0.041	0.008	0.083	0.007	6.937	0.010	0.010	0.947
14	0.253	0.015	nd		0.081	0.005	0.569	0.010	nd		nd		0.009	0.002	0.019	0.002	0.096	0.004	2.131	0.003	0.003	1.025
14.5	0.296	0.015	nd		0.068	0.005	0.526	0.010	nd		nd		0.012	0.008	0.022	0.004	0.093	0.003	1.961	0.003	0.003	1.018
15	0.300	0.013	nd		0.068	0.004	0.516	0.009	nd		nd		0.014	0.002	0.020	0.002	0.088	0.003	1.659	0.002	0.002	1.006
15.5	0.346	0.013	nd		0.068	0.004	0.471	0.009	nd		nd		0.013	0.002	0.019	0.002	0.097	0.003	1.665	0.002	0.002	1.014
16.1	0.617	0.057	nd		0.047	0.017	0.126	0.035	nd		0.037	0.029	0.039	0.030	0.116	0.015	0.040	0.013	19.961	0.036	0.031	1.022
16.4	0.297	0.028	nd		0.117	0.009	0.319	0.018	nd		0.019	0.014	0.073	0.015	0.033	0.008	0.076	0.006	6.592	0.009	0.009	0.934
17.1	0.358	0.014	nd		0.037	0.005	0.460	0.009	nd		nd		0.024	0.002	0.028	0.002	0.084	0.003	1.898	0.003	0.003	0.990
18	0.580	0.029	0.099	0.031	0.168	0.014	nd		nd		0.030	0.005	nd		0.051	0.005	nd		14.705	0.021	0.023	0.929
18.9	0.309	0.041	nd		0.089	0.013	0.302	0.027	nd		nd		0.099	0.006	0.070	0.006	0.093	0.010	15.474	0.022	0.020	0.962
20	nd		nd		0.252	0.027	0.571	0.038	nd		nd		0.101	0.012	0.074	0.014	0.067	0.015	73.540	0.106	0.066	1.063
20.5	0.053	0.028	nd		0.234	0.009	0.485	0.018	nd		0.013	0.014	0.111	0.015	0.063	0.008	0.115	0.006	6.560	0.009	0.006	1.075
23.5	0.368	0.022	nd		0.026	0.007	0.517	0.014	nd		nd		0.015	0.003	0.043	0.003	0.097	0.005	4.378	0.006	0.005	1.067

nd = not detected

Supplementary Table 3: Model Parameters

Constraints from Cariaco Basin		***Lit. value***	***1σ for fit***	***Notes***	***Source***
f0 (export production)	--> A	100%		Compares long term (2002 – 2010) mean OC fluxes in sub-photic zone	Muller-Kargar et al., 2001;
C flux (vs. f0)	A --> B	51%	15%	("Z", 53 mgC/m2/d) and near-sediment traps ("D", 27 gC/m2/d) to	Werne et al., 2003; Thunell
	B --> C	38%	5%	equivalent sediment TOC accumulations at 5–10 cm (~20 mgC/m2/d) and 185–275 cm (~16 mgC/m2/d).	and Tappa, 2010; Raven et al., 2016
	C --> final	30%	8%		
OM S:C (mol/mol)	A --> B	*poorly constrained*			--
	B --> C	2.0%	0.5%		Werne et al., 2003
	C --> final	3.7%	0.5%		Werne et al., 2003

Assigned parameter ranges		***min.***	***max.***	***Sensitivity tests (Supp. Fig. 3)***	***Continuous-G formulation***	***Source***
turnover time				***"shorter residence times"***		
	A	3.5 days	21 days	1 – 10 days	v_z = 2.5 E4 – 1.5 E5 m/yr; Z = 1400 m	Muller-Kargar et al., 2001; 2004
	B	2 yrs	500 yrs	1 – 50 yrs	v_z = 1.5 E–4 to 2.5 E–3 m/yr; Z = 5 E–3 m	Boudreau, 2003 (bioturbation to 10 cm depth at 0.2 mm/yr)
	C	2,000 yrs	20,000 yrs	2,000 – 20,000 yrs (no change)	v_z = 1.5 E–4 to 2.5 E–4 m/yr; Z = 10 m	Werne et al., 2001
effective S:C ratio				***"higher / lower S:C for rapid sulf."***		
	biomass	0%	1%	0 – 1% (no change)		Francois et al., 1987
	rapid sulf (A+B)	4%	5%	3.5–4.5% (lower S:C); 5–6% (higher S:C)		Hetzel et al., 2009; Van Kaam–Peters et al., 1998; etc.
	gradual sulf (B+C)	1%	3%	1 – 3% (no change)		moieties in recalacitrant organics
"juiciness" or relative reactivity				***"Wider range of OM reactivity classes"***		
	J1	1.0E+00		1.0E+00 (no change)		
	J2	1.0E-02		1.0E-02 (no change)		
	J3	1.0E-04		1.0E-05 (allows 10x faster initial OM reactivity)		Werne et al., 2001

Supplementary References

1. Muller-Karger, F. *et al.* Processes of coastal upwelling and carbon flux in the Cariaco Basin. *Deep Sea Research Part II: Topical Studies in Oceanography* **51,** 927–943 (2004).
2. Raven, M. R., Sessions, A. L., Adkins, J. F. & Thunell, R. C. Rapid organic matter sulfurization in sinking particles from the Cariaco Basin water column. *Geochimica et Cosmochimica Acta* **190,** 175–190 (2016).
3. Gelin, F., Kok, M. D., De Leeuw, J. W. & Damsté, J. S. S. Laboratory sulfurisation of the marine microalga Nannochloropsis salina. *Organic Geochemistry* **29,** 1837–1848 (1998).
4. Rowland, S., Rockey, C., Al-Lihaibi, S. S. & Wolff, G. A. Incorporation of sulphur into phytol derivatives during simulated early diagenesis. *Organic Geochemistry* **20,** 1–5 (1993).
5. Pohlabeln, A. Marine dissolved organic sulfur – Sources, fate, and structural characteristics. 1–171 (2017).
6. Controls on the molecular and carbon isotopic composition of organic matter deposited in a Kimmeridgian euxinic shelf sea: evidence for preservation of carbohydrates through sulfurisation. *Geochimica et Cosmochimica Acta* **62,** 3259–3283 (1998).
7. Hetzel, A., Böttcher, M. E., Wortmann, U. G. & Brumsack, H.-J. Paleo-redox conditions during OAE 2 reflected in Demerara Rise sediment geochemistry (ODP Leg 207). *Palaeogeography, Palaeoclimatology, Palaeoecology* **273,** 302–328

(2009).

8. Kolonic, S. *et al.* Geochemical characterization of Cenomanian/Turonian black shales from the Tarfaya Basin (SW Morocco). *Journal of Petroleum Geology* **25,** 325–350 (2002).

9. Eglinton, T. I., Irvine, J. E., Vairavamurth, Zhou, W. & Manowitz, B. Formation and diagenesis of macromolecular organic sulfur in Peru margin sediments. *Organic Geochemistry* **22,** 781–799 (1994).

10. Johnson, S. G. The NLopt nonlinear optimization package.

11. Webb, S. M. SIXpack: a graphical user interface for XAS analysis using IFEFFIT. *Phys. Scr.* **2005,** 1011 (2005).
